# Neuroendocrine Differentiation in Breast Cancer: Clinicopathological Significance of Bcl-2 Positive Solid Papillary Carcinoma

**DOI:** 10.1155/2016/9501410

**Published:** 2016-12-25

**Authors:** Yoichiro Okubo, Takuji Okubo, Yoshimi Okubo, Takao Ishiwatari

**Affiliations:** ^1^Department of Pathology, Kanagawa Cancer Center, 2-3-2 Nakao, Asahi-Ku, Yokohama, Kanagawa 241-8515, Japan; ^2^Department of Internal Medicine, Okubo Internal Medicine/Surgery Clinic, 1282 Ohaza, Oita, Oita Prefecture 870-1151, Japan; ^3^Department of Surgery, Okubo Internal Medicine/Surgery Clinic, 1282 Ohaza, Oita, Oita Prefecture 870-1151, Japan; ^4^Department of Pathology, Chiba Cytopathological Center, 5-27-8 Nishifuna, Funabashi, Chiba 273-0031, Japan

## Abstract

Solid papillary carcinoma (SPC) is considered a rare malignant breast tumor. Maluf and Koerner first reported this disease entity as a special type of ductal carcinoma in situ with several characteristic histopathological features, including low-grade cellular atypia, intracellular or extracellular mucin deposition, and solid papillary growth pattern, as well as neuroendocrine differentiation. The present paper describes a case of SPC with bcl-2 expression, which is known as a marker for malignancy of neuroendocrine tumors. Interestingly, despite bcl-2 expression being a poor prognostic indicator of neuroendocrine tumors, the patient with this tumor has achieved long-term survival (approximately 6 years) at the time of writing this report. Because previous investigators reported that bcl-2 expression might play a role in the inhibition of the development of breast cancer, we suggest that bcl-2 expression might reflect a good prognosis in patients with SPC, rather than being a poor prognostic indicator, as it is in several types of neuroendocrine tumor. However, to confirm this hypothesis, further investigation is required.

## 1. Introduction

Solid papillary carcinoma (SPC) is considered a rare malignant breast tumor, with an incidence ranging from 1.1% to 1.7% of all malignant breast tumors [1–10]. Maluf and Koerner first reported this disease entity as a special type of ductal carcinoma in situ (DCIS) with several characteristic histopathological features, including low-grade cellular atypia, intracellular or extracellular mucin deposition, and solid papillary growth pattern, as well as neuroendocrine differentiation [2]. Furthermore, the 2010 World Health Organization (WHO) classification categorized this tumor as a solid papillary carcinoma (ICD-O codes: 8509/2 or 8509/3, with and without invasion) and indicated that solid neuroendocrine carcinoma originated from SPC [11]. The present paper describes a case of SPC with low Ki-67 labeling index, absence of p53 expression, and bcl-2 expression, which is known as a marker for malignancy of neuroendocrine tumors [12–20].

## 2. Case Presentation

**Figure 1:**
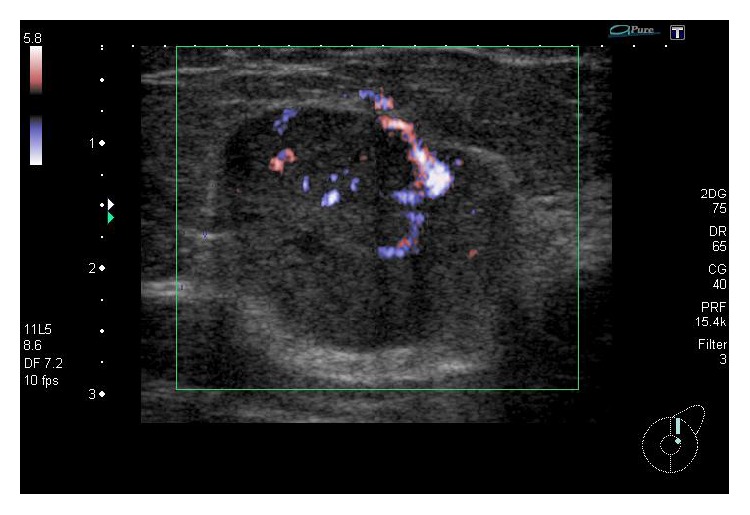
A breast ultrasonography image shows well-demarcated, highly abundant blood flow and a cystic lesion. These findings suggest a breast tumor with malignancy.

**Figure 2:**
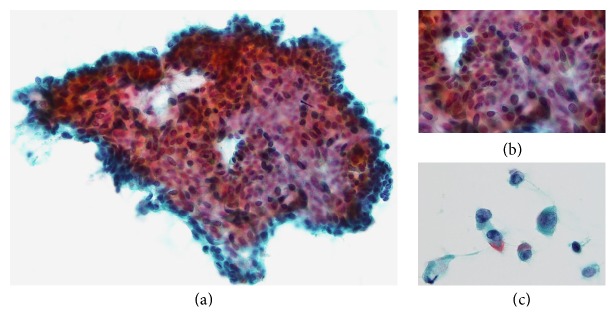
(a) Tumor cells form a papillary and conglomerate mass. Individual tumor cells have a round to short spindle-shaped nucleus with granular chromatin (Papanicolaou stain, original magnification ×400). (b) Individual tumor cells have a round to short spindle-shaped nucleus with granular chromatin. Nuclear atypia is not prominent. Some tumor cells show ill-defined rosette formation (Papanicolaou stain, original magnification ×1000). (c) Some tumor cells remain as separate cells rather than forming a mass. Many of these tumor cells contain cytoplasmic mucin. There are no prominent nucleoli (Papanicolaou stain, original magnification ×1000).

A 62-year-old Japanese woman presenting with a mass in the left breast was admitted to Japanese hospital for evaluation of this lesion. Mammography showed a high-density mass, and breast ultrasonography showed well-demarcated, highly abundant blood flow and a cystic lesion (Figure 1). Because these results indicated the presence of a malignant tumor, fine-needle aspiration cytology and needle biopsy were performed. Aspiration cytology indicated a lesion suspicious for carcinoma (Figure 2). If tumor cells show ill-defined rosette formation, these findings indicate solid papillary carcinoma. But actually this is not 100% proven. We therefore conducted needle biopsy that suggested ductal carcinoma. Consequently, the patient underwent partial mastectomy of the left breast with lymph node dissection. Our patient's surgical intervention had a successful outcome—neither local recurrence nor distant metastasis occurred, and she maintains a high quality of life 70 months after the operation.

**Figure 3:**
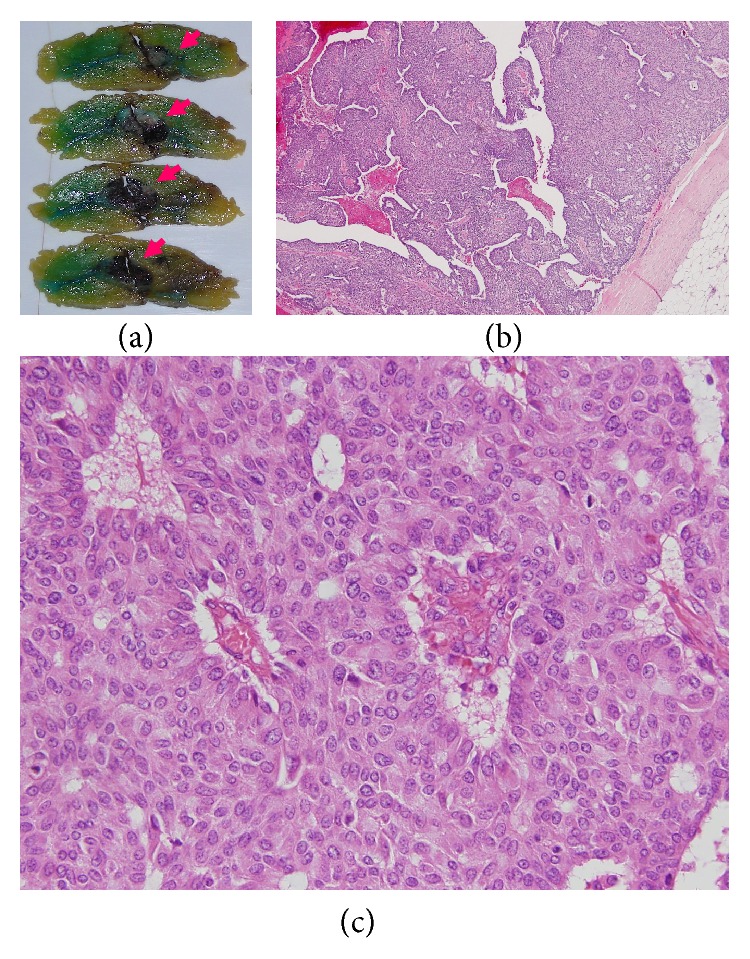
(a) A solid tumor 25 × 30 × 25 mm in size whose surface is sharply demarcated from the surrounding tissue (arrow) is seen at the cut surface of the specimen. Both necrosis and hemorrhage are present. (b) Sections of paraffin-embedded tissue were prepared and stained with HE double stain for light microscopic observation. A histological section reveals intraductal carcinoma showing a solid papillary growth pattern and the neoplastic lesion is encapsulated by bands of dense fibrosis (HE stain, original magnification ×40). (c) Nuclear palisading around fibrovascular cores was found (HE stain, original magnification ×400).

**Figure 4:**
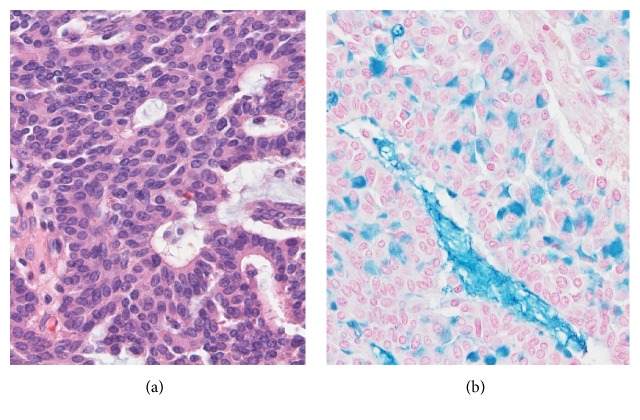
(a) The tumor cells have a round to elongated and hyperchromatic nucleus plus finely granular eosinophilic cytoplasm. In addition, rosette formation is seen (HE stain, original magnification ×400). (b) Intracellular and extracellular mucin deposition (focal distribution) is present (Alcian blue stain, original magnification ×400).

The surgical specimens, resected breast tissue and lymph nodes, were fixed in 10% buffered formalin. A solid tumor 25 × 30 × 25 mm in size and sharply demarcated from the surrounding tissue was visible at the cut surface of the breast tissue (Figure 3). Both necrosis and hemorrhage were present. Sections of paraffin-embedded tissue were prepared and stained with hematoxylin and eosin (HE) stain for light microscopic observation. Histological examination revealed intraductal carcinoma showing a solid papillary growth pattern and nuclear palisading around fibrovascular cores (Figure 3). The neoplastic lesion was encapsulated by bands of dense fibrosis. The tumor cells had a round to elongated and hyperchromatic nucleus plus finely granular eosinophilic cytoplasm. Intracellular and extracellular mucin deposition was present (Figure 4). Neither lymphovascular involvement nor lymph node metastasis was present.

**Figure 5:**
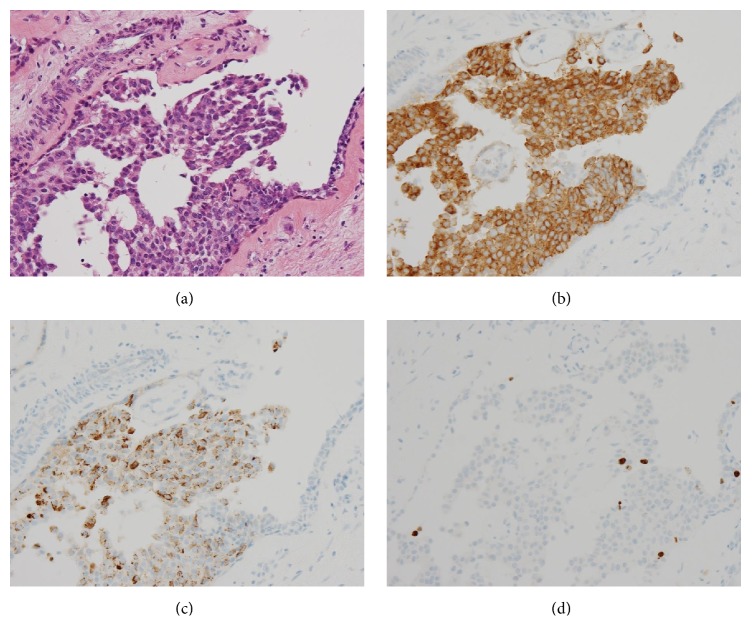
(a) The tumor cells show a solid papillary growth pattern (HE stain, original magnification ×400). (b) The tumor cells show positive immunoreactivity for synaptophysin (Immunohistochemistry, original magnification ×400). (c) The tumor cells show positive immunoreactivity for chromogranin A (Immunohistochemistry, original magnification ×400). (d) A few tumor cells show positive immunoreactivity for Ki-67 (Immunohistochemistry, original magnification ×400).

**Figure 6:**
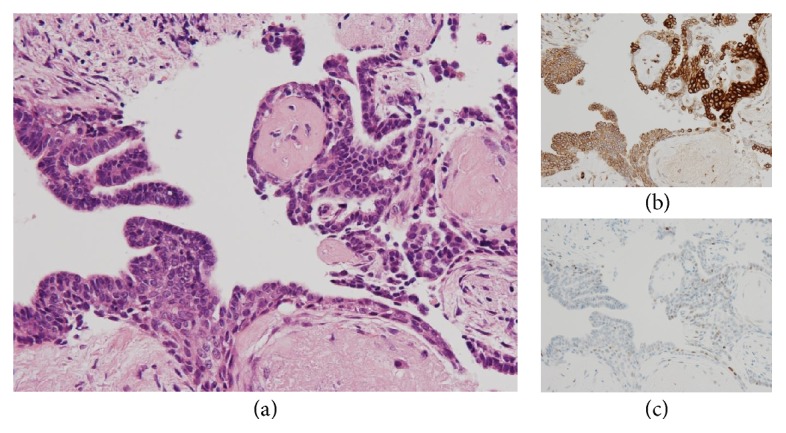
(a) The tumor cells show papillary growth pattern and nuclear palisading around fibrovascular cores (HE stain, original magnification ×400). (b) The tumor cells show positive immunoreactivity for bcl-2 (Immunohistochemistry, original magnification ×400). (c) A few tumor cells weakly show positive immunoreactivity for p53 (Immunohistochemistry, original magnification ×400). (d) A few tumor cells show positive immunoreactivity for Ki-67 (Immunohistochemistry, original magnification ×400).

Various monoclonal antibodies were used to evaluate the tumor cells immunohistochemically, including antisynaptophysin, chromogranin A, estrogen receptor (ER), progesterone receptor (PgR), human epidermal growth factor receptor 2 (HER-2), bcl-2, p53, and Ki-67 antibodies. The tumor cells showed positive immunoreactivity for synaptophysin, chromogranin A, ER, PgR, and bcl-2 (Figures 5 and 6) and negative (or almost negative) immunoreactivity for HER-2. The Ki-67 labeling index was 3.7% and almost negative or weak immunoreactivity for p53 was found (Figures 5 and 6). In the present study, representative antibodies against the following were then used via immunohistochemically evaluating the tumor cells: bcl-2 (1 : 50 dilution; Dako Japan, Tokyo, Japan, Clone name: 124), chromogranin A (1 : 800 dilution; Dako Japan, Clone name: DAK-A3), Ki-67 (1 : 200 dilution; Dako Japan, Clone name: MIB-1), and synaptophysin (1 : 40 dilution; Dako Japan, Clone name: M0776).

## 3. Discussion

Although SPC was first reported by Maluf and Koerner in 1995 [2], this disease entity remains underrecognized by clinicians, cytotechnologists, and pathologists [1]. As previously mentioned, the 2010 WHO classification categorized this tumor as a solid papillary carcinoma. It is well known that SPC usually arises in the seventh or eighth decade and has a better prognosis than other breast cancers [11]. Histopathologically, the tumor cells show a solid papillary growth pattern, nuclear palisading around fibrovascular cores, and a round to elongated nucleus plus finely granular eosinophilic cytoplasm [1]. Immunohistochemically, tumor cells usually show positive reactivity for neuroendocrine markers, including synaptophysin and chromogranin A. In addition, these tumors generally show positive reactivity for ER and PgR [1]. Several types of low-grade ductal carcinomas should be considered as differential diagnoses for SPC. In particular, papillary carcinoma is similar to SPC in many respects. Namely, it arises in elderly patients, grows in large well-circumscribed ducts, and is characterized by a good prognosis [5]. However, this tumor differs from SPC by the presence of delicate papillae, a branching pattern, a cuboidal to columnar appearance of the tumor cells, and the lack of a solid growth pattern [5, 11]. Certain benign intraductal tumors should be considered because of their solid or papillary intraductal epithelial growth pattern [5, 11]. However, a uniform cell population, increased mitotic activity, nuclear palisading around fibrovascular cores, and intracellular or extracellular mucin deposition are not usually seen in benign intraductal tumors [5, 7, 11]. Furthermore, the importance of the confirmation of neuroendocrine differentiation has often been described [5]. In the present paper, the tumor cells followed the characteristic features of SPC, including a solid papillary growth pattern, nuclear palisading around fibrovascular cores, a round to elongated and hyperchromatic nucleus, finely granular eosinophilic cytoplasm, and intracellular or extracellular mucin deposition, as well as positive reactivity for synaptophysin, but without positive reactivity for chromogranin A.

The immunohistochemical examination findings of this tumor are worthy further discussion. Ki-67 is known as a prognostic indicator of neuroendocrine tumors [12, 17, 21, 22]. Kawasaki et al. reported that the Ki-67 labeling index of nonneuroendocrine DCIS (8.1%) was significantly higher than that of neuroendocrine DCIS (4.3%), and they concluded that a low Ki-67 labeling index is correlated with a good prognosis in patients with neuroendocrine DCIS [20]. In fact, our patient showed an exceptionally low Ki-67 labeling index (3.7%). In addition, several studies on neuroendocrine tumors have reported that bcl-2 or p53 expression might be correlated with malignant behavior [13–17, 19, 23–27]. However, no studies were found in which investigation was limited to SPC. Therefore, we tried to establish the immunohistochemical prognostic indicators of SPC by using bcl-2 and p53, which are acceptable prognostic indicators in several types of neuroendocrine tumor. As a result, the tumor cells showed negative reactivity for p53, which may reflect a good prognosis in patients with SPC. However, the tumor cells showed positive reactivity for bcl-2, which is a poor prognostic indicator of neuroendocrine tumors [17].

To explain this paradoxical result, we considered the meaning of bcl-2 expression in breast cancer. Won et al. reported that beclin-1 and bcl-2 expression might play a role in the inhibition of the development of breast cancer [28]. Therefore, we suggested that bcl-2 expression also reflexes the good prognosis of the patients with SPC rather than prognostic indicators in several kinds of neuroendocrine tumor. Unfortunately, our immunohistochemical examinations are based on only one patient with SPC. To confirm this hypothesis, further investigation is required.
